# Hypertrophic Cardiomyopathy Cardiac Troponin C Mutations Differentially Affect Slow Skeletal and Cardiac Muscle Regulation

**DOI:** 10.3389/fphys.2017.00221

**Published:** 2017-04-20

**Authors:** Tiago Veltri, Maicon Landim-Vieira, Michelle S. Parvatiyar, David Gonzalez-Martinez, Karissa M. Dieseldorff Jones, Clara A. Michell, David Dweck, Andrew P. Landstrom, P. Bryant Chase, Jose R. Pinto

**Affiliations:** ^1^Department of Biomedical Sciences, Florida State University College of MedicineTallahassee, FL, USA; ^2^Department of Molecular and Cellular Pharmacology, University of Miami Miller School of MedicineMiami, FL, USA; ^3^Section of Pediatric Cardiology, Department of Pediatrics, Baylor College of MedicineHouston, TX, USA; ^4^Department of Biological Science, Florida State UniversityTallahassee, FL, USA

**Keywords:** cardiac troponin C, hypertrophic cardiomyopathy, slow skeletal muscle, skinned fibers, myofibrillar ATPase

## Abstract

Mutations in *TNNC1*—the gene encoding cardiac troponin C (cTnC)—that have been associated with hypertrophic cardiomyopathy (HCM) and cardiac dysfunction may also affect Ca^2+^-regulation and function of slow skeletal muscle since the same gene is expressed in both cardiac and slow skeletal muscle. Therefore, we reconstituted rabbit soleus fibers and bovine masseter myofibrils with mutant cTnCs (A8V, C84Y, E134D, and D145E) associated with HCM to investigate their effects on contractile force and ATPase rates, respectively. Previously, we showed that these HCM cTnC mutants, except for E134D, increased the Ca^2+^ sensitivity of force development in cardiac preparations. In the current study, an increase in Ca^2+^ sensitivity of isometric force was only observed for the C84Y mutant when reconstituted in soleus fibers. Incorporation of cTnC C84Y in bovine masseter myofibrils reduced the ATPase activity at saturating [Ca^2+^], whereas, incorporation of cTnC D145E increased the ATPase activity at inhibiting and saturating [Ca^2+^]. We also tested whether reconstitution of cardiac fibers with troponin complexes containing the cTnC mutants and slow skeletal troponin I (ssTnI) could emulate the slow skeletal functional phenotype. Reconstitution of cardiac fibers with troponin complexes containing ssTnI attenuated the Ca^2+^ sensitization of isometric force when cTnC A8V and D145E were present; however, it was enhanced for C84Y. In summary, although the A8V and D145E mutants are present in both muscle types, their functional phenotype is more prominent in cardiac muscle than in slow skeletal muscle, which has implications for the protein-protein interactions within the troponin complex. The C84Y mutant warrants further investigation since it drastically alters the properties of both muscle types and may account for the earlier clinical onset in the proband.

## Introduction

Hypertrophic cardiomyopathy (HCM) is a cardiac disease with relatively high prevalence (1:200) in the general population, which promotes morphological changes of the heart such as left ventricular thickening (Semsarian et al., 2015). The first case of HCM was described in a patient from the 1950's (Teare, 1958) in which the diagnosis of HCM development was solely based on electrocardiogram analyses associated with angiographic/hemodynamic studies. After 1990, the development of DNA-based sequencing methodologies provided a valuable resource which accelerated a new era of HCM diagnosis with the discovery of the first mutation in the ß-myosin heavy chain gene, *MYH7* (Geisterfer-Lowrance et al., 1990). Currently, it is widely accepted that mutations in sarcomeric genes lead to intracellular alterations that manifest as cardiac remodeling and pathologic hypertrophy (Force et al., 2010; Marian, 2010; Harvey and Leinwand, 2011; Seidman and Seidman, 2011; Maron and Maron, 2013).

After two decades of extensive genetic investigation and over 1,400 mutations identified, many genes encoding thin and thick filament, and cytoskeletal proteins have been established as the etiological agents of HCM (Maron et al., 2012). The most common mutations are located in thick-filament encoding genes (*MYH7* and *MBPC3*), representing ~50–70% of all HCM patients that exhibit positive genetic test results. Meanwhile, mutations in genes that encode thin-filament proteins, such as the troponin (Tn) complex, have also been shown to occur with relatively high incidence compared to other canonical sarcomeric HCM genes (Willott et al., 2010; Brouwer et al., 2011; Maron et al., 2012). HCM-associated mutations lead to several alterations in cardiac contraction e.g., changes in myosin ATPase activity, actomyosin crossbridge interaction, cooperativity of muscle activation, Ca^2+^ binding to the thin filament, and a host of other HCM predisposing factors (Willott et al., 2010). In general, mutations in the regulatory proteins (tropomyosin and Tn) that are linked to HCM are associated with an increase in myofilament Ca^2+^ sensitivity; thus promoting changes in Ca^2+^ homeostasis and triggering development of cardiac dysfunction and arrhythmias seen in HCM patients (Willott et al., 2010; Brouwer et al., 2011; Landstrom and Ackerman, 2012).

Limited studies of HCM patients' skeletal muscle have identified abnormalities in electromyographic (EMG) and/or histologic analyses (Hootsmans and Meerschwam, 1971; Smith et al., 1976; Lochner et al., 1981; Przybojewski et al., 1981). In 1989, Caforio et al. (1989) showed that skeletal muscle dysfunction occurred in 37% of HCM patients analyzed and found that this dysfunction arose from myogenic origins. This finding was independently verified in skeletal muscle biopsies. The authors also observed selective atrophy of type I muscle fibers in these patients (Caforio et al., 1989). Moreover, a recent clinical study which examined 46 HCM patients by EMG showed evidence of subclinical skeletal muscle myopathy in 13 of the patients (28%) (Karandreas et al., 2000).

Many genes for cardiac sarcomeric proteins are also expressed in some skeletal muscles; these include ß-myosin heavy chain (*MYH7*), regulatory myosin light chain-2 (*MYL2*), desmin (*DES*), and troponin C (*TNNC1*) (Li et al., 1989; Matsuoka et al., 1989; Macera et al., 1992; Song et al., 1996). Mutations in the *MYH7* gene can produce simultaneous abnormalities in cardiac and slow-twitch skeletal muscle function that can culminate as cardiomyopathy and/or myopathy (Hedera et al., 2003; Tajsharghi et al., 2003, 2007; Meredith et al., 2004; Mastaglia et al., 2005; Lamont et al., 2006, 2014; Darin et al., 2007; Overeem et al., 2007; Homayoun et al., 2011). Similarly, the *MYL2* gene is expressed in both skeletal and cardiac muscle; mutations in myosin light chain appear to affect the function and morphology of both tissue types (Weterman et al., 2013). Desmin, a sarcomeric protein and a primary component of most intermediate filaments, is also a target of muscle disease. Desmin is expressed in cardiac, skeletal, and smooth muscle, and many studies have shown that mutations in the human *DES* gene promote adult-onset skeletal myopathy and, depending on which mutation, one of the three types of familial cardiomyopathies, i.e., dilated, hypertrophic, and restrictive (Goldfarb and Dalakas, 2009).

Troponin C (TnC) is a key regulatory protein in striated muscle contraction where its function is to bind Ca^2+^, which subsequently triggers actomyosin interactions and initiates muscle contraction (Farah and Reinach, 1995). The *TNNC1* gene expresses TnC in both cardiac and slow skeletal muscle (Song et al., 1996). Seven mutations in *TNNC1* (A8V, L29Q, A31S, C84Y, E134D, D145E, and Q122AfsX30) have been identified to date in HCM and restrictive cardiomyopathy patients (Hoffmann et al., 2001; Landstrom et al., 2008; Chung et al., 2011; Parvatiyar et al., 2012; Jaafar et al., 2015; Ploski et al., 2016). The A8V mutation was genetically knocked in and led to HCM in mice (Martins et al., 2015). Unfortunately, no clinical data regarding skeletal muscle abnormalities is available for these patients. With the exception of the E134D mutant, *in vitro* studies indicate that the above mentioned cTnC mutants alter contractile parameters known to underlie the development of diastolic dysfunction in these patients (Landstrom et al., 2008; Pinto et al., 2009, 2011a; Albury et al., 2012; Parvatiyar et al., 2012; Zot et al., 2016). Despite their extensive characterization in cardiac muscle, however, there are no reports on the effects of these HCM-associated *TNNC1* mutations on slow skeletal muscle regulation and function.

The purpose of these *in vitro* studies was to identify potential pathogenic alterations in slow skeletal muscle regulation arising from the cTnC mutants. Therefore, we evaluated the effects of cTnC A8V, C84Y, E134D, and D145E mutants associated with HCM on the Ca^2+^ sensitivity and maximal force when reconstituted into skinned fibers from rabbit soleus, and maximal and minimal ATPase activities when reconstituted into bovine masseter muscle myofibrils. These muscle types were chosen for this study because they are type I fibers that only express myosin heavy chain I (MHC I) and show sufficient resilience after isolation that is suitable for each type of assay presented. Human TnC was utilized to accentuate translational value of the study. The primary sequence of cTnC is the same among human, porcine and bovine homologs, and rabbit cTnC differs by only one amino acid. Other Tn subunits including slow skeletal troponin I (ssTnI) homologs are largely similar (close to 99% homologous) among human, bovine, porcine, and rabbit. However, it should be noted that rabbit slow skeletal troponin T (ssTnT) isoforms are less homologous to human, porcine, and bovine homologs. Utilization of proteins from different but overall largely homologous species has been widely accepted, although small functional differences cannot be discounted. Overall, we found that these cTnC mutants induce mutation-dependent effects in slow skeletal muscle that were distinct from their effects in cardiac muscle; at least part of that distinction was found to depend on the TnI isoform. Our results are consistent with findings from other major muscle proteins encoded by the same gene in both cardiac and skeletal muscles. The data presented in this manuscript will be discussed in the context of the patients' clinical presentation.

## Materials and methods

### SDS-PAGE for myosin heavy chain (MHC) separation

Skeletal muscle MHC composition was analyzed by glycerol-containing SDS-PAGE. Eight percent acrylamide gels were prepared and run according to Talmadge and Roy (1993). Protein concentration was measured using the Pierce™Coomassie Plus (Bradford) Assay Kit (Bradford, 1976). Myofibrils and skeletal myosin (0.5–1 μg) were boiled in sample buffer (Laemmli, 1970) for 2 min and loaded into glycerol-containing gels. 2-mercaptoethanol was added to the upper electrode buffer at a final concentration of 10 mM. Gels were run at constant voltage (75 V) in a Bio-Rad Mini-PROTEAN Tetra System for 27 h at 8°C. After, gels were stained with silver nitrite.

### Protein expression and purification

Human cTnC (WT and mutants), ssTnI, and cardiac troponin T (cTnT) were expressed in *E. coli* and purified as described previously (Landstrom et al., 2008; Pinto et al., 2008a).

### Formation of binary complex ssTnI.cTnC

Five different binary complexes (ssTnI.cTnC-WT, ssTnI.cTnC-A8V, ssTnI.cTnC-C84Y, ssTnI.cTnC-E134D, and ssTnI.cTnC-D145E) were assembled essentially as described previously (Pinto et al., 2008a) for use in displacement assays. After expression and purification, the individual human troponin subunits of ssTnI or cTnC (WT or mutants) were first dialyzed against a buffer containing high urea (3M) and KCl (1M), then against two changes of buffer with the same salt composition but without urea. After this step, the protein concentration was determined using the Pierce^*TM*^ Coomassie Plus (Bradford) Assay Kit. Then ssTnI and individual cTnC (WT, A8V, C84Y, E134D, or D145E) proteins were mixed in a molar ratio of 1.3:1 ssTnI:cTnC. The protein mixtures were dialyzed against buffers of decreasing KCl concentration until binary complexes were formed (ssTnI-cTnC). Next, the proteins were dialyzed into relaxing solution (pCa 8.0 = 10^−8^ M free [Ca^2+^], 1 mM free [Mg^2+^], 7 mM EGTA, 2.5 mM MgATP^2−^, 20 mM MOPS, pH 7.0, 20 mM creatine phosphate, ionic strength was adjusted to *I* = 0.15 M using potassium propionate at 21°C). Protein aggregates were removed by centrifugation and the binary complexes were stored at −80°C until use.

### Skinned fibers

Slow skeletal skinned fibers were isolated from rabbit soleus muscle. New Zealand White rabbits were sacrificed and muscles were isolated in accordance with NIH guidelines and animal care protocol approved by the Animal Care and Use Committees of University of Miami and Florida State University. Soleus muscles were tied to toothpicks and excised at the resting, *in vivo* length from the experimental animals. After this, the slow skeletal fibers were immersed in relaxing low Ca^2+^ solution containing EGTA and 1% V/V Protease Inhibitor Cocktail (Sigma-Aldrich, P8340) (for details, see Pinto et al., 2008b).

For skinned cardiac preparations, left ventricular papillary muscles were dissected from fresh porcine hearts that were obtained from a local abattoir. Small muscle bundles were dissected and incubated overnight in relaxing low Ca^2+^ solution containing 1% Triton X-100 at 4°C to remove membranes.

Both slow skeletal and cardiac preparations were stored at −20°C in relaxing solution (pCa 8.0) plus glycerol (52% V/V). For mechanical measurements, isolated slow skeletal fibers or cardiac muscle preparations were attached to a force transducer on one end and a microtranslator on the other end to adjust the length of the muscle preparation, and immersed initially in relaxing solution (pCa 8.0 with 15 units/ml creatine phosphokinase added). Muscle preparations were stretched at pCa 8.0 by 20% over slack length. Initial, maximal Ca^2+^-activated force of slow skeletal fibers and cardiac preparations was tested in saturating Ca^2+^ conditions (pCa 4.0 = 10^−4^ M free [Ca^2+^]) where the solution composition was otherwise the same as described above for relaxing solution.

### Extraction of endogenous TnC from slow skeletal skinned fibers and reconstitution with WT or HCM mutant cTnC

After the determination of initial, maximal, active force (P_0_), the endogenous TnC was depleted by exposing slow skeletal skinned fibers to a CDTA extraction solution for fibers (5 mM 1,2-cyclohexylenedinitrilotetraacetic acid (CDTA), pH 8.4 adjusted with dried Tris powder) for 1.5–2 h, as described previously for extraction of TnC from skinned cardiac preparations (Landstrom et al., 2008). After successive washings with relaxing solution to remove CDTA, the residual active force at pCa 4.0 was measured to determine the efficacy of TnC extraction. The average residual force values after extraction were 22.4 ± 1.6, 25.3 ± 0.8, 25.1 ± 0.8, 23.7 ± 1.8, 26.0 ± 0.6% of P_0_ for fibers that were to be reconstituted with WT, A8V, C84Y, E134D, or D145E, respectively. The residual force after extraction was not statistically different (ANOVA and *t*-test) among the five groups of fibers. For reconstitution with cTnC, the soleus fibers were successively exposed to drops of solution containing 28 μM cTnC, either WT or one of the mutants, in pCa 8.0 solution. TnC extraction and reconstitution were carried out at room temperature. To test the efficacy of TnC reconstitution, maximal force was tested again at pCa 4.0 for comparison with P_0_.

### Displacement of endogenous cTn complex from cardiac preparations and reconstitution with binary complex ssTnI.cTnC-WT or ssTnI.cTnC-HCM mutant

After measuring P_0_, the skinned cardiac preparations were equilibrated for 10 min in pre-incubation buffer (250 mM KCl, 20 mM MOPS, 5 mM MgCl_2_, 5 mM EGTA, 1 mM DTT, pH 6.2) (Pinto et al., 2008a) without cTnT. The fibers were then treated for 2.5 h with ~0.8 mg/ml cTnT in the same buffer. The cTnT-treated fibers were subsequently washed in pre-incubation buffer without added cTnT to remove unbound, endogenous Tn complex and excess exogenous cTnT. The amount of endogenous Tn complex displaced by the excess cTnT was measured as the percentage of Ca^2+^-unregulated force, %UF = ([tension at pCa 8.0/tension at pCa 4.0]*100) (Pinto et al., 2008a). Subsequently, the fibers were incubated in pCa 8.0 buffer containing pre-formed binary complex ssTnI.cTnC WT or mutant) for 1 h until the Ca^2+^-regulated force was restored (i.e., the fibers relaxed) and had reached steady state. All procedures were carried out at room temperature.

### Ca^2+^ dependence of force development and cooperativity of slow skeletal fibers containing cTnC mutants or cardiac preparations containing ssTnI.cTnC mutants

Measurement of Ca^2+^ sensitivity and cooperativity of thin filament activation of steady-state, isometric force were performed using slow skeletal skinned fibers (reconstituted with WT or mutant cTnC) or skinned cardiac preparations (reconstituted with ssTnI.cTnC binary complexes containing WT or HCM mutants). The reconstituted slow skeletal or cardiac preparations were incubated in sequentially increasing [Ca^2+^] solutions, ranging from pCa 8.0 to 4.0 (decreasing pCa). The standard buffer for Ca^2+^ sensitivity curves was 1 mM free [Mg^2+^], 7 mM EGTA, 2.5 mM MgATP^2−^, 20 mM MOPS (pH 7.0), 20 mM creatine phosphate, and 15 units/ml creatine phosphokinase, I = 150 mM and free [Ca^2+^] range from 10^−8^ to 10^−4^ M (pCa 8 to pCa 4). A pCa calculator program by Dweck et al. (2005) was used to calculate free [Ca^2+^] in the pCa solutions. Force measurements were conducted at room temperature (~21°C). Steady-state, isometric force data were normalized and fit to the 4-parameter Hill equation (Hill, 1910):

$$\begin{array}{l} P=\frac{P_{0}{\left[Ca^{2+}\right]}^{n_{Hill}}}{{\left[Ca^{2+}\right]}^{n_{Hill}}+{\left[Ca_{50}^{2+}\right]}^{n_{Hill}}}+P_{min} \end{array}$$

where *P*_0_ is the maximal force, *P* is the normalized force, “[${\text{Ca}}_{50}^{2+}$]” is the free [Ca^2+^] that produces 50% force and the exponent “*n*_Hill_” is the Hill coefficient, which is related to the steepness of the relationship around [${\text{Ca}}_{50}^{2+}$] and is an indicator of apparent cooperativity for Ca^2+^ activation of steady-state force. The [${\text{Ca}}_{50}^{2+}$] parameter estimates obtained from regression are reported as pCa_50_ (i.e., −log[${\text{Ca}}_{50}^{2+}$].

### Myofibril preparation

**Figure 1:**
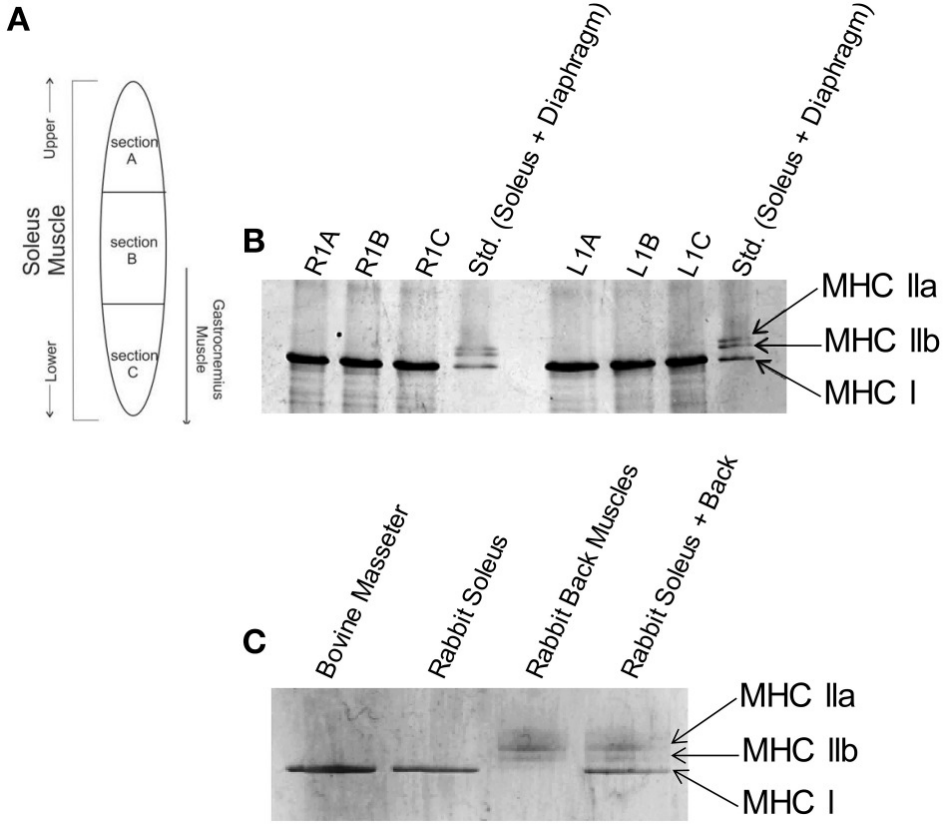
**Slow skeletal myosin heavy chain I is the predominant isoform throughout rabbit soleus and bovine masseter muscles. (A)** Schematic of locations where muscle samples were collected from rabbit soleus muscle to determine whether there is a spatial gradient of different fiber types. **(B)** Glycerol SDS-PAGE shows that only the slow skeletal myosin isoform (MHC I) is detectable throughout rabbit soleus muscle. Gel lanes are annotated as R1A-C or L1A-C to denote the right and left hind leg and sections A, B or C (labels in panel **A**) of the muscle. The standard (Std.) lanes contain rabbit soleus and diaphragm myofibrils, combined, to visualize all major skeletal muscle MHC isoforms. **(C)** Glycerol SDS-PAGE shows that MHC I is the only myosin isoform detectable in bovine masseter muscle myofibrils. Rabbit soleus myofibrils and rabbit back muscles myosin were used as standards for the visualization of other MHC isoforms.

Rabbit soleus and diaphragm myofibrils used in Figure 1 were prepared as described previously (Solaro et al., 1971) and stored in 50% glycerol at –20°C. Rabbit back muscles myosin used in Figure 1 was prepared as described (Margossian and Lowey, 1982) and stored in 50% glycerol at −20°C. Myofibrils were prepared from porcine cardiac (used in **Figure 3**) and bovine masseter (used in Figures 1, 3) muscle, obtained from a local abattoir, as described previously (Solaro et al., 1971) and stored in 50% glycerol at −20°C. For SDS-PAGE analyses of MHC composition, rabbit soleus, rabbit diaphragm, bovine masseter myofibrils, and rabbit back muscles myosin from glycerol stocks were washed with MF buffer (30 mM Imidazole, 60 mM KCl, 2 mM MgCl_2_, pH 7.0, 1V myofibril or myosin/14V MF buffer). For myofibrillar ATPase activity assays, glycerol was removed by re-suspending and centrifuging the myofibrils using myofibril wash buffer containing 10 mM MOPS, 10 mM KCl, 2 mM dithiothreitol (DTT), pH 7.0 (1V myofibril/5V wash buffer x 3).

### Myofibrillar TnC extraction and reconstitution

Native TnC was depleted from myofibrils by incubating in CDTA extraction buffer for myofibrils (5 mM CDTA, 5 mM DTT, pH 8.4 adjusted with dried Tris powder) for ~2.5 h at room temperature. Every 30 min, the sample was centrifuged and the supernatant was discarded. At the end, myofibrils were washed three times in wash buffer to remove CDTA. Myofibrils were then reconstituted with cTnC–WT or—HCM mutants for 1 h at 4°C. In order to minimize non-specific binding of cTnC, the reconstituted samples were washed several times with myofibril wash buffer. Quantification of myofibril protein concentration was performed using the Pierce™Coomassie Plus (Bradford) Assay Kit. Native, TnC-extracted, and TnC reconstituted myofibrils at 0.26 mg/ml were boiled in sample buffer (Laemmli, 1970) for 2 min and loaded into a 15% SDS-polyacrylamide gel.

### Myofibrillar ATPase activity

Myofibrillar ATPase assays were performed using a buffer containing 2 mM EGTA, 3 mM nitrilotriacetic acid (NTA), 20 mM MOPS, 1 mM free Mg^+2^, ~2.5 mM MgATP^2−^, pH = 7.0 with a fixed *I* = 80 mM and constant myofibril concentration of 0.4 mg/ml at 25°C. Solutions were either pCa 8.0 or pCa 5.0. A pCa calculator program by Dweck et al. (2005) was used to calculate free [Ca^2+^] in the pCa solutions. The reaction was initiated by adding 2.94 mM ATP and quenched after 7 min by adding 4.6% trichloroacetic acid. After the assay reaction was terminated, precipitated proteins were removed by centrifugation for 7 min, at 3,095 × g and 4°C. The concentration of inorganic phosphate in the supernatant, released by ATP hydrolysis, was measured using a colorimetric method according to the method of Fiske and Subbarow (1925).

### Statistical analyses

Data were tested for significant differences using Student's *t*-test (unpaired) and ANOVA with *post-hoc* Tukey's Honest Significant Difference (HSD) test. For Student's *t*-test, mutant protein data were always compared to WT within the same experimental condition. For ANOVA, a multiple comparison approach was used. Differences for all statistical tests were considered significant when *p* < 0.05. When ANOVA yielded significance, Tukey's HSD test was then used to identify pairwise differences, and we report those differences found between mutant proteins vs. WT. Formally, multiple comparison using ANOVA is the statistically appropriate starting point, to be followed by a *post-hoc* test where significance was found by ANOVA. We have also included the results of pairwise comparisons using Student's *t*-tests where significance was found for consistency with prior work on cardiac muscle where only *t*-test analyses were performed. Our general interpretation is that ANOVA/Tukey's HSD is the more stringent test that identifies the strongest significant differences (which were also identified by *t*-tests), while those identified by *t*-test (alone) provide guidance for changes that may still prove to be relevant. The data were expressed as mean ± S.E.M. Non-linear least squares regressions and Student's *t*-test analyses were performed using SigmaPlot software (version 12.0). ANOVA analyses and *post-hoc* Tukey's HSD tests were performed using R (version 3.3.2).

## Results

The effects of HCM-associated cTnC mutants on the regulation of cardiac myofilaments have been extensively characterized. In Figure 1A, the schematic indicates the location of which muscle sections were excised from rabbit soleus muscle in order to assess whether different MHC isoforms are expressed within the sections. Figure 1B shows glycerol SDS-PAGE analysis of MHC isoforms in myofibrils isolated from rabbit soleus muscles, illustrating that only the slow-skeletal myosin isoform (i.e., MHC I) was uniformly detectable throughout these muscles. Glycerol SDS-PAGE analysis in Figure 1C shows, similarly, that only MHC I was detectable in bovine masseter muscle myofibrils.

**Figure 2:**
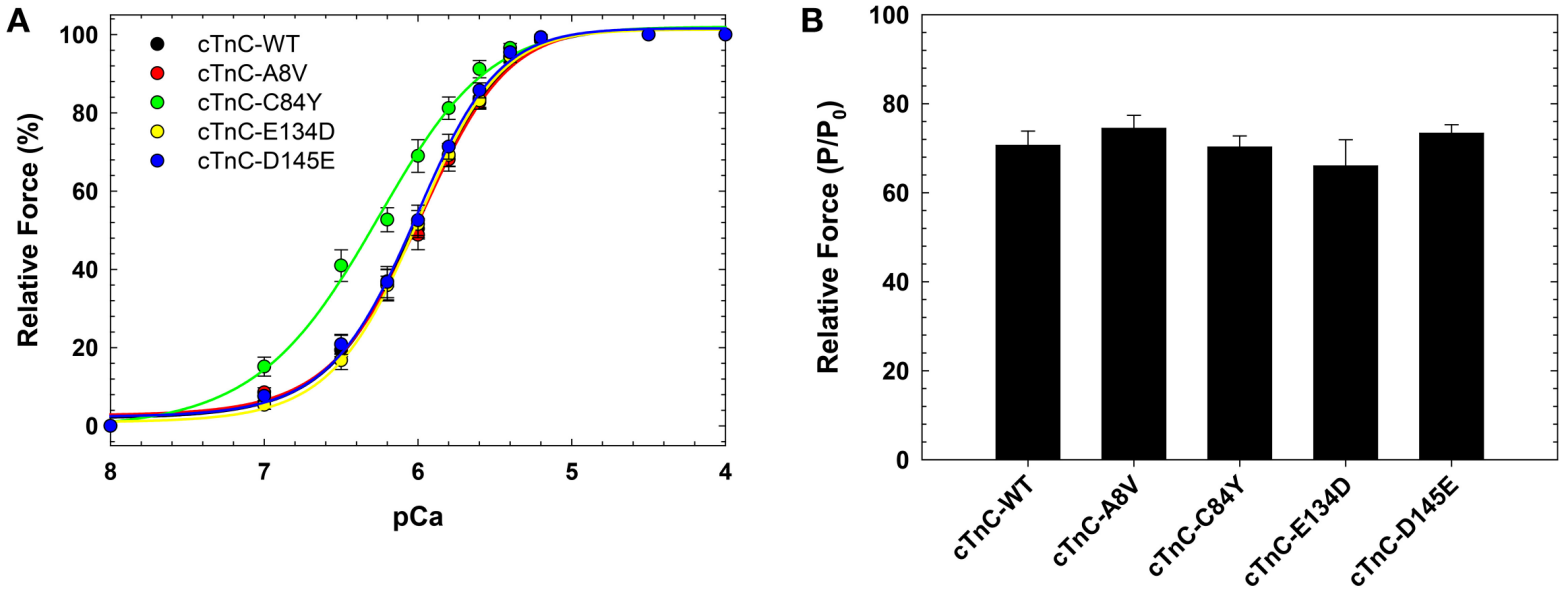
**Ca^**2+**^ dependence of steady-state, isometric force development in rabbit soleus fibers reconstituted with recombinant cTnC proteins. (A)** Ca^2+^ sensitivity of contraction in skinned fibers reconstituted with WT and HCM cTnC mutants (A8V, C84Y, E134D, and D145E). **(B)** Maximal force (pCa 4) following reconstitution normalized to the initial tension (P/P_0_) in fibers reconstituted with WT or HCM cTnC mutants. Regression parameter estimates of pCa_50_ and *n*_Hill_, along with ΔpCa_50_ and relative maximal force values for these experiments are summarized in Table 1. Data are shown as mean ± S.E.M.

We previously showed that cTnC A8V, C84Y, and D145E mutants increased Ca^2+^ sensitivity of steady-state, isometric force development when reconstituted into skinned cardiac preparations, while the cTnC E134D mutant did not affect Ca^2+^ sensitivity (Table 1) (Landstrom et al., 2008). Here, we determined the effects of the HCM cTnC mutants on the regulation of slow skeletal fibers. To accomplish this, TnC-depleted skinned fibers from rabbit soleus muscle were reconstituted with one of the exogenous cTnC proteins (WT, A8V, C84Y, E134D, or D145E). Only the C84Y mutant displayed a significant increase in the Ca^2+^ sensitivity of force development and a decrease of the *n*_Hill_ (*t*-test and ANOVA/Tukey HSD, Figure 2A and Table 1). The pCa_50_ (i.e., -log free [Ca^2+^] which yields a half-maximal response, where [Ca^2+^] is in molar units) obtained for cTnC C84Y reconstituted slow skeletal fibers was 6.33 ± 0.07; whereas, the pCa_50_ of fibers reconstituted with WT cTnC was 6.00 ± 0.03. Moreover, when comparing the Ca^2+^-sensitization associated with the cTnC C84Y mutant between reconstituted slow skeletal and cardiac fibers, the slow skeletal muscle fibers further accentuated this effect. This difference is reflected in the ΔpCa_50_ (difference between the pCa_50_ of fibers reconstituted with cTnC C84Y and WT) of slow skeletal muscle (+0.33 log units) and cardiac muscle (+0.27 log units; Landstrom et al., 2008) (Table 1). The *n*_Hill_ for slow skeletal fibers containing cTnC C84Y was significantly lower than WT, 1.26 ± 0.05 vs. 1.61 ± 0.07 (Figure 2A and Table 1).

**Table 1:** **Parameter summary for Ca^**2+**^ dependence of steady-state isometric force in skinned porcine cardiac and rabbit soleus muscle preparations reconstituted with WT cTnC or cTnC HCM mutants**.

**Muscle**	**TnC (WT and mutants)**	**pCa_50_**	**ΔpCa_50_**	***n*_Hill_**	**Maximal force (%)**	**# Experiments**
Slow Skeletal	WT	6.00 ± 0.03		1.61 ± 0.07	70.65 ± 3.22	10
	A8V	6.01 ± 0.04	+0.01	1.65 ± 0.09	74.47 ± 2.96	16
	C84Y	6.33 ± 0.07^*^^,^^#^	+0.33	1.26 ± 0.05^*^^,^^#^	70.26 ± 2.51	11
	E134D	6.01 ± 0.03	+0.01	1.68 ± 0.09	66.00 ± 5.92	8
	D145E	6.05 ± 0.04	+0.05	1.63 ± 0.08	73.35 ± 1.94	11
Cardiac^a^	WT	5.66 ± 0.01		2.74 ± 0.19	59.1 ± 2.3	8
	A8V	6.02 ± 0.01^*^	+0.36	2.68 ± 0.18	72.4 ± 2.7^*^	9
	C84Y	5.93 ± 0.01^*^	+0.27	2.42 ± 0.15^*^	59.5 ± 3.7	8
	E134D	5.68 ± 0.01	+0.02	2.82 ± 0.16	58.4 ± 2.1	7
	D145E	5.90 ± 0.01^*^	+0.24	2.73 ± 0.17	70.3 ± 1.4^*^	8

a *Values from Landstrom et al. (2008)*.

*ΔpCa_50_ = pCa_50_ of mutant TnC–pCa_50_ of WT TnC*.

* *p < 0.05 HCM mutant vs. WT tested with Student's t-test*.

# *p < 0.05 from ANOVA and p < 0.05 from post-hoc Tukey's HSD test for HCM mutant vs. WT*.

Although reconstitution of the cTnC A8V or D145E mutants into skinned cardiac fibers led to an increase in the Ca^2+^ sensitivity of contraction (Landstrom et al., 2008), this was not observed in the slow skeletal fibers (compared to WT cTnC reconstituted soleus fibers; Figure 2A and Table 1), suggesting that the presence of slow skeletal proteins could play a crucial role in normalizing the Ca^2+^ sensitivity of contraction. The *n*_Hill_ values of soleus muscle fibers reconstituted with cTnC A8V or D145E were not statistically different from the WT control, indicating that in slow skeletal muscle, these cTnC mutants do not alter cooperativity (Table 1).

Reconstitution of the cTnC E134D mutant did not alter the Ca^2+^ sensitivity or *n*_Hill_ of either skinned cardiac or slow skeletal preparations (Figure 2A and Table 1). Additionally, no significant differences in the maximal force were observed among slow skeletal fibers that were reconstituted with the WT or any of the four HCM cTnC mutants (Figure 2B and Table 1).

**Figure 3:**
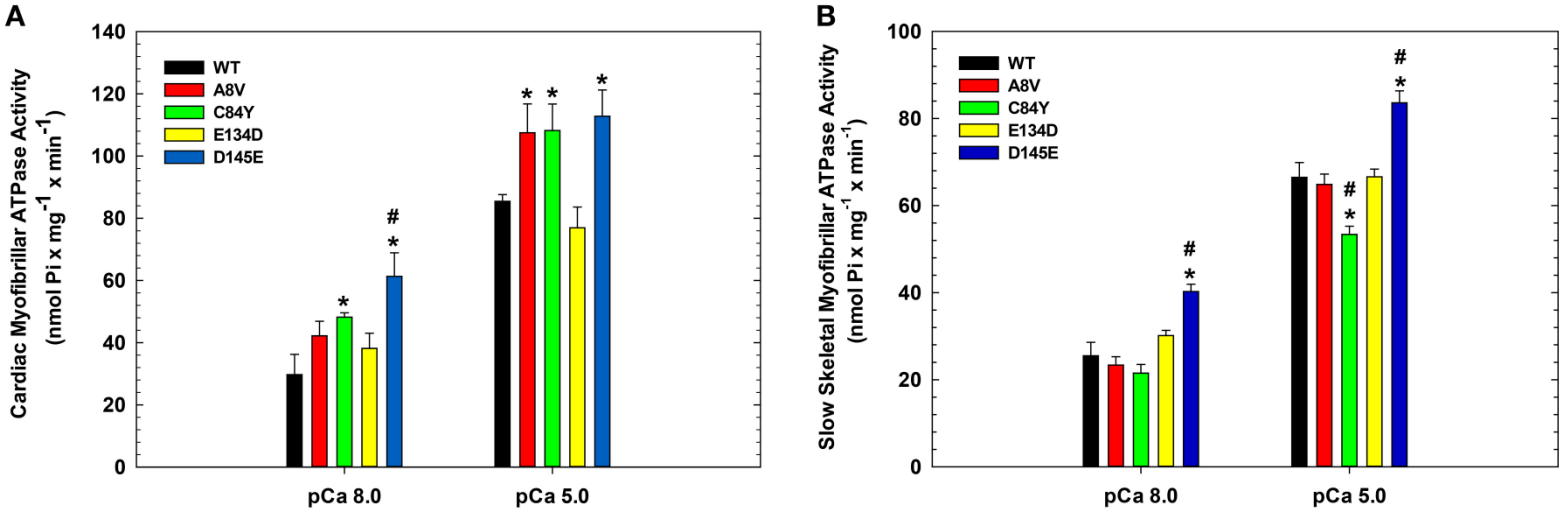
**Myofibrillar ATPase activity of (A)** porcine cardiac and **(B)** bovine masseter muscle myofibrils that were depleted of TnC and reconstituted with WT cTnC or cTnC HCM mutants. (^*^) Indicates significant differences (*p* < 0.05, Student's *t*-test) for mutant cTnCs compared to WT in the same muscle type and pCa. (^#^) Indicates significant differences (*p* < 0.05, ANOVA/Tukey's HSD) for mutant cTnCs compared to WT in the same muscle type and pCa. ATPase values for these experiments are summarized in Table 2. Data are shown as mean ± S.E.M.

To assess the effects of the cTnC mutants on myofibrillar ATPase activity, we performed assays comparing the ATPase activity of both cardiac and slow skeletal myofibrils reconstituted with recombinant WT cTnC or mutants. Supplementary Figure 1 shows the SDS-PAGE analysis of cardiac and slow skeletal myofibrils, demonstrating TnC extraction from native myofibrils followed by cTnC reconstitution. At inhibiting (sub-diastolic) Ca^2+^ concentration (pCa 8.0), cardiac myofibrils reconstituted with cTnC D145E (*t*-test and ANOVA/Tukey's HSD), or C84Y and D145E (*t*-tests) displayed significantly higher activities than cardiac myofibrils reconstituted with the WT control (Figure 3A and Table 2). This suggests that the cTnC D145E mutant, and possibly also the C84Y mutant, modifies inhibitory processes within cardiac muscle at low levels of Ca^2+^. At maximal activating Ca^2+^ (pCa 5.0), cardiac myofibrils reconstituted with cTnC A8V, C84Y, or D145E exhibited higher levels of myofibrillar ATPase activity when individually compared to the WT control (*t*-tests), although no significant differences were identified by ANOVA (Figure 3A and Table 2). Differences in muscle regulation by the cTnC mutants were especially evident for the C84Y and D145E mutants in slow skeletal myofibrils. In the slow skeletal muscle background, cTnC C84Y decreased the myofibrillar ATPase activity at maximally activating Ca^2+^ (pCa 5.0) when compared to the WT control (*t*-test and ANOVA/Tukey HSD; Figure 3B and Table 2). The effect of reconstituting the D145E mutant in slow skeletal myofibrils showed similar trends in ATPase activity to those observed in cardiac myofibrils: the D145E mutant increased ATPase activity at both low and high Ca^2+^ concentrations when compared to the WT control (*t*-test and ANOVA/Tukey HSD; Figure 3B and Table 2).

**Table 2:** **Summary of Ca^**2+**^ dependent ATPase activities from porcine cardiac and bovine masseter myofibrils reconstituted with WT cTnC or cTnC HCM mutants**.

	**WT**	**A8V**	**C84Y**	**E134D**	**D145E**
**CARDIAC MYOFIBRILS**					
Min. ATPase activity (pCa 8.0)	29.70 ± 6.52	42.19 ± 4.67	48.20 ± 1.39^*^	38.14 ± 4.87	61.33 ± 7.58^*^^,^^#^
Max. ATPase activity (pCa 5.0)	85.44 ± 2.17	107.50 ± 9.29^*^	108.20 ± 8.55^*^	76.94 ± 6.67	112.83 ± 8.41^*^
**SLOW SKELETAL MYOFIBRILS**					
Min. ATPase activity (pCa 8.0)	25.50 ± 3.10	23.35 ± 1.91	21.50 ± 2.01	30.13 ± 1.22	40.25 ± 1.67^*^^,^^#^
Max. ATPase activity (pCa 5.0)	66.50 ± 3.41	64.84 ± 2.37	53.38 ± 1.89^*^^,^^#^	66.63 ± 1.76	83.63 ± 2.72^*^^,^^#^

*Values are shown as nmol Pi x mg^−1^ x min^−1^*.

Min = minimum and Max = maximum

*Native cardiac myofibrils ATPase activity: 16.80 ± 0.58 and 67.60 ± 1.43 at pCa 8.0 and 5.0, respectively. TnC-extracted cardiac myofibrils ATPase activity 7.20 ± 2.01 and 8.80 ± 2.43 at pCa 8.0 and 5.0, respectively*.

*Native slow skeletal myofibrils ATPase activity: 46.75 ± 1.25 and 82.50 ± 1.18 at pCa 8.0 and 5.0, respectively. TnC-extracted cardiac myofrils ATPase activity 13.40 ± 0.92 and 33.40 ± 2.31 at pCa 8.0 and 5.0, respectively*.

* *p < 0.05 HCM mutant vs. WT tested with Student's t-test*.

# *p < 0.05 from ANOVA and p < 0.05 from post-hoc Tukey's HSD test for HCM mutant vs. WT*.

*Cardiac myofibrils, n = 5–7*.

*Slow skeletal myofibrils, n = 8–14*.

Although cTnC A8V and D145E are capable of altering the physiological properties of cardiac muscle contraction (e.g., ΔpCa_50_ of +0.36 and +0.24, respectively), these mutants did not substantially alter the corresponding properties of slow skeletal muscle (Table 1). The presence of slow skeletal thin filament proteins may have counteracted the enhancement of apparent Ca^2+^ affinity at cTnC's N-terminal regulatory Ca^2+^ binding site which was observed in the cardiac preparations reconstituted with cTnC A8V or D145E. To better understand the influence of the different sarcomeric isoforms (i.e., cardiac vs. slow skeletal) on these phenomena, we tested whether these effects are partially or totally attributed to the presence of ssTnI. Therefore, we exchanged the endogenous cTn complex of skinned cardiac fibers with exogenous chimeric Tn complexes comprised of cTnC, ssTnI, and cTnT. To assess the amount of displaced endogenous cTn achieved by the cTnT displacement method, we recorded the average values of the cardiac preparation's Ca^2+^-unregulated force (i.e., force generated by preparations that have displaced cTnC and cTnI by incubation with cTnT), which were: 88.5 ± 3.4, 82.1 ± 4.2, 80.2 ± 7.2, 86.7 ± 8.4, and 91.7 ± 3.8% for preparations that were to be reconstituted with cTnC WT, A8V, C84Y, E134D, or D145E, respectively. After cTn displacement, cardiac preparations were incubated with the exogenous binary complex containing ssTnI and WT or mutant cTnC. Finally, the recovered maximal Ca^2+^ regulated force was recorded. The maximal force values were: 81.1 ± 4.7, 82.7 ± 4.6, 91.8 ± 2.4, 92.1 ± 5.0, and 84.4 ± 4.1% of the P_0_ for WT, A8V, C84Y, E134D, and D145E, respectively. No significant differences in the Ca^2+^-unregulated force, as well as, the Ca^2+^-regulated maximal force among the five experimental groups existed (*t*-test and ANOVA; Table 3).

**Table 3:** **Parameter summary for Ca^**2+**^ dependence of steady-state isometric force in skinned porcine cardiac preparations reconstituted with ssTnI together with WT cTnC or cTnC HCM mutants**.

**ssTnI.cTnC (WT and mutants)**	**pCa_50_**	**ΔpCa_50_**	***n*_Hill_**	**Maximal force (P/Po)%**	**% Ca^2+^ unregulated force**	**# Experiments**
WT	5.73 ± 0.02	–	1.74 ± 0.04	81.09 ± 4.69	88.51 ± 3.38	8
A8V	5.82 ± 0.01^*^^,^^#^	+0.09	1.70 ± 0.02	82.68 ± 4.64	82.09 ± 4.16	8
C84Y	6.14 ± 0.03^*^^,^^#^	+0.41	1.28 ± 0.04^*^^,^^#^	91.83 ± 2.41	80.20 ± 7.15	7
E134D	5.73 ± 0.03	–	1.71 ± 0.11	92.10 ± 5.02	86.67 ± 8.43	5
D145E	5.91 ± 0.01^*^^,^^#^	+0.18	1.57 ± 0.03^*^	84.42 ± 4.11	91.67 ± 3.80	6

*The Ca^2+^ unregulated force following TnT incubation (to displace endogenous troponin) was calculated according to: (P_pCa8_/P_pCa4_) X 100, where P_pCa8_ and P_pCa4_ are the forces generated at pCa 8.0 and pCa 4.0, respectively*.

*ΔpCa_50_ = pCa_50_ of mutant TnC–pCa_50_ of WT TnC*.

* *p < 0.05 HCM mutant vs. WT tested with Student's t-test*.

# *p < 0.05 from ANOVA and p < 0.05 from post-hoc Tukey's HSD test for HCM mutant vs. WT*.

**Figure 4:**
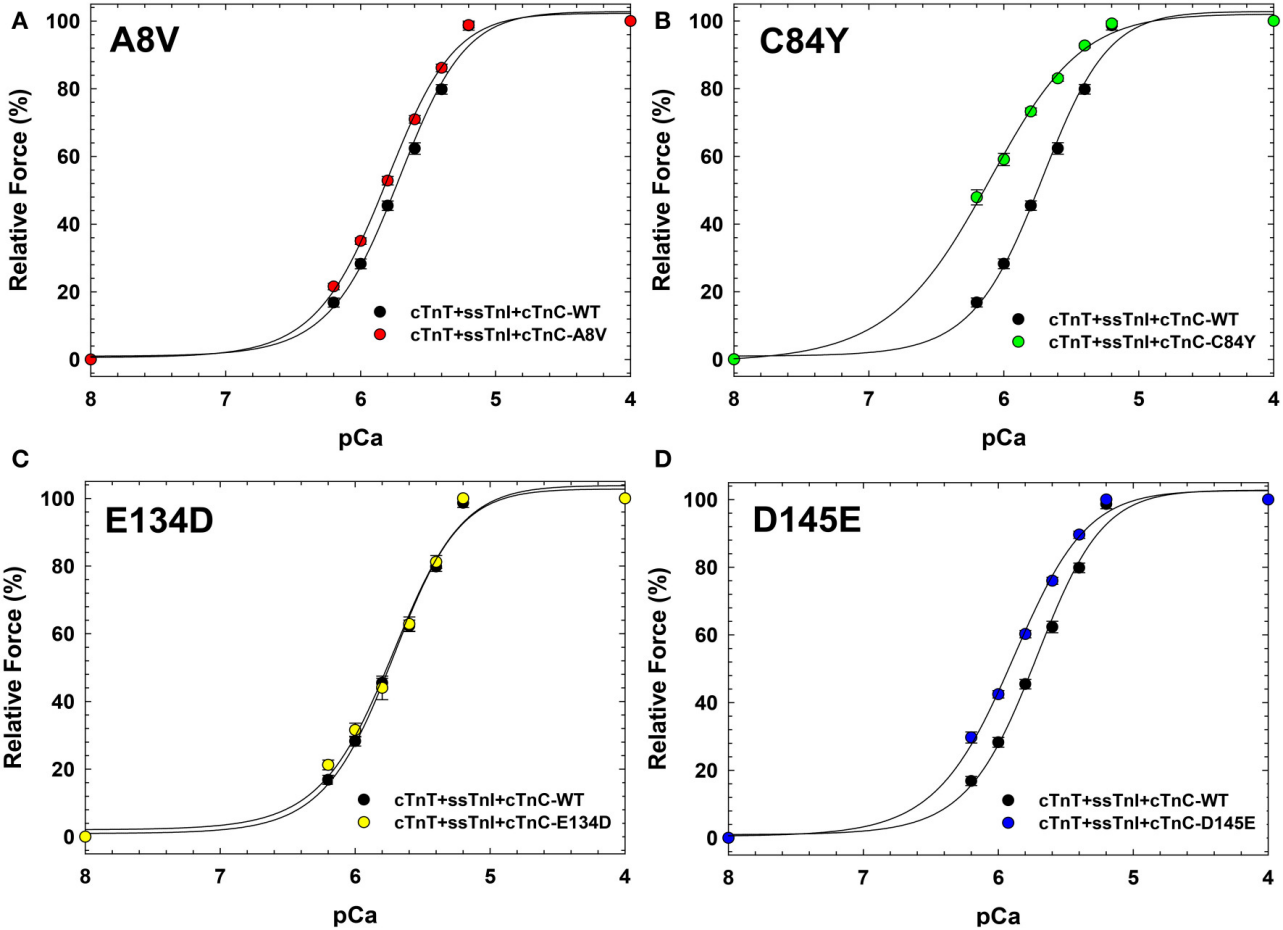
**Ca^**2+**^ dependence of steady-state isometric force development in porcine cardiac preparations reconstituted with slow skeletal TnI together with WT cTnC or cTnC HCM mutants**. Comparisons of Ca^2+^ sensitivity of force development obtained for cTnC WT and HCM mutants, in **(A)** WT vs. A8V, **(B)** WT vs. C84Y, **(C)** WT vs. E134D, and **(D)** WT vs. D145E. Regression parameter estimates of pCa_50_ and *n*_Hill_, along with ΔpCa_50_ and relative maximal force values for these experiments are summarized in Table 3. Data are shown as mean ± S.E.M.

Figure 4 indicates that incorporation of ssTnI into cardiac preparations partially attenuated the effects of cTnC A8V and D145E on the Ca^2+^ sensitivity of force development. The ΔpCa_50_ values for cardiac preparations reconstituted with cTnC A8V and D145E together with ssTnI were +0.09 and +0.18, respectively (Figure 4 and Table 3); whereas, in skinned cardiac preparations reconstituted with cTnC A8V and D145E alone (i.e., in the presence of endogenous cTnI), the ΔpCa_50_ values were +0.36 and +0.24, respectively (Table 1) (Landstrom et al., 2008). In contrast, in skinned cardiac preparations reconstituted with binary complex containing ssTnI and cTnC C84Y, the presence of ssTnI did not attenuate the myofilament Ca^2+^ sensitization that was observed in the presence of endogenous cTnI. These cardiac preparations exhibited an even higher Ca^2+^ sensitivity of force development than what was observed in cardiac preparations containing the cardiac isoform of TnI (ΔpCa_50_ +0.41 or +0.27, respectively; Tables 1, 3). A comparison of *n*_Hill_ regression parameter estimates obtained from skinned cardiac preparations reconstituted with ssTnI and HCM cTnC mutants indicates that the C84Y (*t*-test and ANOVA/Tukey's HSD) and D145E (*t*-test) mutants significantly reduced apparent cooperativity in comparison to the WT control (1.28 ± 0.04, 1.57 ± 0.03, and 1.74 ± 0.04 for C84Y, D145E, and WT, respectively; Table 3). The cardiac preparations reconstituted with exogenous ssTnI and cTnC E134D showed no significant changes in relation to the pCa_50_ and *n*_Hill_ (*t*-test and ANOVA/Tukey's HSD) when compared with WT control (Figure 4 and Table 3).

## Discussion

Patients that exhibit cardiomyopathy and possess a positive family history are routinely screened for mutations in a number of thin and thick filament encoding genes. Obvious clinical symptoms such as dyspnea, syncope, chest pain, and arrhythmia—in addition to characteristic cardiac remodeling revealed through imaging—are frequently documented complaints. Many sarcomeric mutations that are identified on the basis of cardiac dysfunction, however, are also expressed in skeletal muscle. With the clinical focus on the heart, more subtle skeletal muscle abnormalities may go undetected (Towbin, 2014). We are interested in whether these mutations may have pathogenic consequences in skeletal muscle that go unreported and/or undetected. Here, we queried whether mutations in *TNNC1* that are linked to HCM and expressed in slow skeletal muscle can manifest as a dysfunction or affect the contractile properties of slow skeletal muscle.

While the cardiac effects of *TNNC1* mutants were first examined in the context of HCM pathogenic properties and disease causation, they have not been assessed in slow skeletal muscle. This study is the first to examine the effects of mutations present in the *TNNC1* gene on slow skeletal muscle regulation (Song et al., 1996). We found that for most of the HCM cTnC mutants examined in this study, their incorporation into slow skeletal muscle ablated the increased Ca^2+^ sensitivity of force development that is seen in cardiac preparations. Only cTnC C84Y maintained its Ca^2+^ sensitizing effects in the slow skeletal muscle fibers. Surprisingly, the effects of this mutation were amplified when incorporated into the slow skeletal myofilament. This suggests that this mutant exerts distinct effects on a common element that exist within both muscle types. Since skeletal muscle comprises a large percentage of a person's mass, and slow type I muscle fibers are found throughout human musculature, it is reasonable to expect that slow muscle dysfunction could impart a substantial burden on the affected individual.

Differences in the protein structure of slow skeletal and cardiac TnI and TnT underlie the functional properties of these muscle types, especially when coupled with the distinctive properties of each mutant cTnC in this study. The overall structures of cardiac and skeletal muscle troponins are similar, at least in troponin's core domain (Takeda et al., 2003; Vinogradova et al., 2005). However, three major differences between skeletal and cardiac troponin structures may contribute to the differential effects: (1) structure of the TnI inhibitory segment is ordered in the skeletal muscle isoform but flexible and disordered in the cardiac isoform (Takeda et al., 2003; Vinogradova et al., 2005); (2) the position and conformation of the TnT2 N-terminal helix (Takeda et al., 2003; Vinogradova et al., 2005); and (3) cTnI contains an N-terminal extension—not present in the skeletal isoforms—which harbors two serine residues capable of undergoing phosphorylation by protein kinase A (Zhang et al., 1995). Based upon the above, the experiments involving ssTnI would not be influenced by phosphorylation of the cardiac-specific, N-terminal extention of cTnI.

The myofibrillar ATPase assays further explored the mechanism underlying the Ca^2+^ sensitization induced by the C84Y mutant in both types of muscle fibers. At saturating Ca^2+^ (pCa 5.0), slow skeletal ATPase activity was significantly reduced, while cardiac ATPase activity trended higher. In cardiac muscle, the residue C84 contacts the cTnI switch region when the N-terminus of cTnC is in the open conformation and the BC loop of cTnC when it is in the closed conformation. Therefore, the C84Y mutant may stabilize the open conformation when bound to cTnI (Li and Hwang, 2015) and affect the critically important positioning of the cTnC N-domain (Hwang et al., 2014). Upon binding of divalent cations, the global structure of cTnC becomes more compact (Jayasundar et al., 2014; Badr et al., 2016), which could likely be influenced by mutations in the cTnC linker region. These alterations in the cTnC N-domain may increase the affinity between cTnC-C84Y and the cTnI switch region and ultimately impact initiation of cardiac contraction. The switch region of cTnI (148–158) is immediately flanked by the inhibitory (136–147) and regulatory (161–209) regions, which anchors to actin during diastole (Tripet et al., 1997) and, in doing so, is largely responsible for inhibition of contractility at diastolic Ca^2+^ levels (Meyer and Chase, 2016). Increased affinity between cTnC-C84Y and the cTnI switch region would therefore be expected to reduce interactions between the cTnI inhibitory and regulatory regions during diastole, permitting actomyosin ATPase activity at subthreshold Ca^2+^ concentrations, thereby promoting greater ATPase activation at higher Ca^2+^ concentrations.

Based upon the study by Westfall et al. (1997) on ssTnI-overexpressing cardiomyocytes, the reduction in Ca^2+^ activation threshold in slow skeletal fibers containing cTnC-C84Y (Figure 2 and Table 1), may arise from altered interactions within individual regulatory units of the thin filament (7 actin monomers, 1 tropomyosin, and 1 cTn complex). Weaker interactions between ssTnI and cTnC-C84Y may lower the threshold for Ca^2+^-activated development of tension and reduce cooperativity in cardiac muscle reconstituted with ssTnI. In addition, it is also possible that mutations in troponin differentially modulate the ATPase activity of myosin (Schoffstall et al., 2011; Chalovich, 2012) on top of their effects on Ca^2+^ activation of the thin filament. While Ca^2+^-activation of the cardiac thin filament is more dependent on cross-bridges than skeletal muscle (Gillis et al., 2007), cycling crossbridges at submaximally activating Ca^2+^ concentrations could result in reduced cooperativity in the presence of Ca^2+^-sensitizing mutations (Westfall et al., 1997). The less flexible TnI switch region in skeletal muscle appears to differently affect Ca^2+^ regulation of myofibrillar ATPase activity in the presence of the C84Y mutation in cTnC. The differential effect of C84Y on myofibrillar ATPase activity between tissue types suggests that the C84Y mutation in cTnC could increase Ca^2+^ sensitivity of contraction in both muscles by distinct mechanisms. In cardiac muscle, it may increase the overall number of cycling cross-bridges; while in the slow skeletal muscle, it may decrease the overall number of cycling cross-bridges and enhance the population of strongly bound cross-bridges.

The other HCM cTnC mutants examined in this study are located in distinct structural regions of cTnC. A8V is present in the cTnC N-helix, which plays a key role in defining the orientation of the cTnC N-domain relative to the troponin complex. As described above, alterations in cTnC N-domain orientation can alter the affinity between the cTnC N-domain and the cTnI switch peptide; these changes are likely responsible for increasing the cardiac myofibrillar ATPase activity at saturated Ca^2+^ concentration in this (Figure 3 and Table 2) and prior studies (Zot et al., 2016). However, cTnC-A8V does not affect slow skeletal myofibrillar ATPase measurements or alter cooperativity in cardiac preparations reconstituted with cTnC.ssTnI.cTnT. Functionally, the D145E mutant is distinct; it is located in the C-domain of cTnC and has been shown to increase the Ca^2+^ affinity of site II of isolated cTnC, cTn complex and the thin filament (Pinto et al., 2009). The D145E mutant has also been shown to increase cTnC binding affinity to the cardiac thin filament (Marques et al., 2017). The affinity between the TnI switch region and cTnC may be increased regardless of which TnI isoform is present, as indicated by increased activity of myofibrillar ATPase in both the slow skeletal and cardiac myofibrillar ATPase at both low and high Ca^2+^ concentrations (Figure 3 and Table 2). The D145E mutant did not affect the Ca^2+^ sensitivity of skinned slow skeletal fibers (Figure 2 and Table 1). Therefore, it could be speculated that new interactions/altered affinity between the cTnC D145E C-domain and ssTnT could potentially normalize the sensitizing effects of the mutant, which could be confirmed by peptide binding studies. The E134D mutant did not affect myofibrillar ATPase activity (Figure 3 and Table 2) or Ca^2+^-sensitivity of force development or cooperativity of thin filament activation (Figure 2 and Table 1) in either skeletal or cardiac preparations, further confirming the likelihood that it is a non-pathogenic variant (Landstrom et al., 2008).

The incorporation of ssTnI into cardiac muscle attenuated the dysfunction of myofilaments containing the HCM-associated cTnC mutants A8V and D145E. This suggests that ssTnI plays a protective role in regard to its ability to mitigate effects of disease-causing cTnC mutants in the heart. However, the protective role of ssTnI in cardiac muscle contraction is not a novel topic. We have previously shown that ssTnI has the ability to attenuate the functional consequences of two mutations in cTnT that are associated with restrictive cardiomyopathy (Pinto et al., 2008a, 2011b). Others have shown that the expression of ssTnI in the murine heart has a protective effect on skinned cardiac preparations and left ventricular function during stress conditions, such as acidosis (Wolska et al., 2001; Urboniene et al., 2005). As mentioned above, the presence of ssTnI in cardiac muscle preparation was not able to completely abolish the effect of the cTnC mutants A8V and D145E, as seen in Figure 4 in cardiac preparations reconstituted with ssTnI. This suggests that the presence of ssTnT in slow skeletal muscle may also play a role in rescuing the cTnC mutants' functional phenotype. Major differences exist between the structure of ssTnT and cTnT isoforms, where a large portion of the N-terminus present in cTnT is absent in ssTnT (Pinto et al., 2012). This region of cTnT has been shown to have important roles in regulating Ca^2+^ sensitization and activation of the skinned fibers due to effects on the dynamics of strong TnT-Tm interactions (Gomes et al., 2002; Gollapudi et al., 2012). Therefore, we speculate that some of the protective effect observed in slow skeletal muscle could be arising from the N-terminus of ssTnT.

While the crossover of mutation effects in both tissue types may lead to pathological consequences in both slow skeletal and cardiac muscle, manifestations may present themselves over distinctive time courses and with varying severities. When comparing the age of diagnosis and the symptoms of HCM patients in the cohort study that identified four variants in the *TNNC1* gene, it is interesting to note that the patient bearing the C84Y mutant was diagnosed at the earliest age (8.4 years) and exhibited syncope on exertion (Landstrom et al., 2008). Although syncope is a common event, it can signal more ominous underlying conditions (including HCM and arrhythmogenic right ventricular dysplasia) and outcomes such as sudden death (O'Connor et al., 1999). Alterations in skeletal muscle function may lead to additional downstream consequences that remain unknown. Function may be altered further by globally affecting signaling pathways that influence sarcomeric protein isoform switching and energy consumption. These changes could have profound consequences if the aberrant function arises in multiple muscle types, e.g., the cTnC mutants in slow skeletal muscle—despite slow muscle's fatigue-resistant properties that are associated with differential sensitivity to metabolite levels (Chase and Kushmerick, 1988, 1995) in normal individuals—could also be synergistic with regard to patient fatigue or muscle weakness. The true impact of skeletal and cardiac muscle dysfunction as a result of HCM mutations in the *TNNC1* gene will bear out after examination of more cases within the clinic. Individuals affected by mutations in genes expressed more systemically have the potential to influence the time course and severity of disease; therefore, it is of interest to study the broader effects of mutations that may modify disease outcomes when manifesting in multiple muscle types.

## Author note

Part of this work was presented in the Biophysical Society 56^th^ Annual Meeting in San Diego, California, USA (February, 2012).

## Author contributions

Performed experiments: TV, ML, DG, KJ, CM, and DD. Designed Study: TV, MP, DD, AL, PC, and JP. Analyzed Data: TV, ML, CM, DD, and JP. Wrote/Edited Manuscript: TV, ML, MP, DD, AL, PC, and JP.

### Conflict of interest statement

The authors declare that the research was conducted in the absence of any commercial or financial relationships that could be construed as a potential conflict of interest. The reviewer DWDK and handling Editor declared their shared affiliation, and the handling Editor states that the process nevertheless met the standards of a fair and objective review.

## References

[B1] Albury A. N. Swindle N. Swartz D. R. Tikunova S. B. (2012). Effect of hypertrophic cardiomyopathy-linked troponin C mutations on the response of reconstituted thin filaments to calcium upon troponin I phosphorylation. Biochemistry 51, 3614–3621. 10.1021/bi300187k 22489623 PMC3341542

[B2] Badr M. A. Pinto J. R. Davidson M. W. Chase P. B. (2016). Fluorescent protein-based Ca2+ sensor reveals global, divalent cation-dependent conformational changes in cardiac troponin C. PLoS ONE 11:e0164222. 10.1371/journal.pone.0164222 27736894 PMC5063504

[B3] Bradford M. M. (1976). A rapid and sensitive method for the quantitation of microgram quantities of protein utilizing the principle of protein-dye binding. Anal. Biochem. 72, 248–254. 10.1016/0003-2697(76)90527-3 942051

[B4] Brouwer W. P. van Dijk S. J. Stienen G. J. van Rossum A. C. van der Velden J. Germans T. (2011). The development of familial hypertrophic cardiomyopathy: from mutation to bedside. Eur. J. Clin. Invest. 41, 568–578. 10.1111/j.1365-2362.2010.02439.x 21158848

[B5] Caforio A. L. Rossi B. Risaliti R. Siciliano G. Marchetti A. Angelini C.. (1989). Type 1 fiber abnormalities in skeletal muscle of patients with hypertrophic and dilated cardiomyopathy: evidence of subclinical myogenic myopathy. J. Am. Coll. Cardiol. 14, 1464–1473. 10.1016/0735-1097(89)90383-5 2809005

[B6] Chalovich J. M. (2012). Disease causing mutations of troponin alter regulated actin state distributions. J. Muscle Res. Cell Motil. 33, 493–499. 10.1007/s10974-012-9305-x 22678497

[B7] Chase P. B. Kushmerick M. J. (1988). Effects of pH on contraction of rabbit fast and slow skeletal muscle fibers. Biophys. J. 53, 935–946. 10.1016/S0006-3495(88)83174-6 2969265 PMC1330274

[B8] Chase P. B. Kushmerick M. J. (1995). Effect of physiological ADP concentrations on contraction of single skinned fibers from rabbit fast and slow muscles. Am. J. Physiol. 268, C480–C489. 7864087 10.1152/ajpcell.1995.268.2.C480

[B9] Chung W. K. Kitner C. Maron B. J. (2011). Novel frameshift mutation in Troponin C (TNNC1) associated with hypertrophic cardiomyopathy and sudden death. Cardiol. Young 21, 345–348. 10.1017/S1047951110001927 21262074

[B10] Darin N. Tajsharghi H. Ostman-Smith I. Gilljam T. Oldfors A. (2007). New skeletal myopathy and cardiomyopathy associated with a missense mutation in MYH7. Neurology 68, 2041–2042. 10.1212/01.wnl.0000264430.55233.72 17548557

[B11] Dweck D. Reyes-Alfonso A. Potter J. D. (2005). Expanding the range of free calcium regulation in biological solutions. Anal. Biochem. 347, 303–315. 10.1016/j.ab.2005.09.025 16289079

[B12] Farah C. S. Reinach F. C. (1995). The troponin complex and regulation of muscle contraction. FASEB J. 9, 755–767. 7601340 10.1096/fasebj.9.9.7601340

[B13] Fiske C. H. Subbarow Y. (1925). The colorimetric determination of phosphorous. J. Biol. Chem. 66, 375–400.

[B14] Force T. Bonow R. O. Houser S. R. Solaro R. J. Hershberger R. E. Adhikari B.. (2010). Research priorities in hypertrophic cardiomyopathy: report of a Working Group of the National Heart, Lung, and Blood Institute. Circulation 122, 1130–1133. 10.1161/CIRCULATIONAHA.110.950089 20837938 PMC3070356

[B15] Geisterfer-Lowrance A. A. Kass S. Tanigawa G. Vosberg H. P. McKenna W. Seidman C. E.. (1990). A molecular basis for familial hypertrophic cardiomyopathy: a beta cardiac myosin heavy chain gene missense mutation. Cell 62, 999–1006. 10.1016/0092-8674(90)90274-I 1975517

[B16] Gillis T. E. Martyn D. A. Rivera A. J. Regnier M. (2007). Investigation of thin filament near-neighbour regulatory unit interactions during force development in skinned cardiac and skeletal muscle. J. Physiol. 580, 561–576. 10.1113/jphysiol.2007.128975 17317743 PMC2075566

[B17] Goldfarb L. G. Dalakas M. C. (2009). Tragedy in a heartbeat: malfunctioning desmin causes skeletal and cardiac muscle disease. J. Clin. Invest. 119, 1806–1813. 10.1172/JCI38027 19587455 PMC2701871

[B18] Gollapudi S. K. Mamidi R. Mallampalli S. L. Chandra M. (2012). The N-terminal extension of cardiac troponin T stabilizes the blocked state of cardiac thin filament. Biophys. J. 103, 940–948. 10.1016/j.bpj.2012.07.035 23009843 PMC3433604

[B19] Gomes A. V. Guzman G. Zhao J. Potter J. D. (2002). Cardiac troponin T isoforms affect the Ca2+ sensitivity and inhibition of force development: insights into the role of troponin T isoforms in the heart. J. Biol. Chem. 277, 35341–35349. 10.1074/jbc.m204118200 12093807

[B20] Harvey P. A. Leinwand L. A. (2011). The cell biology of disease: cellular mechanisms of cardiomyopathy. J. Cell Biol. 194, 355–365. 10.1083/jcb.201101100 21825071 PMC3153638

[B21] Hedera P. Petty E. M. Bui M. R. Blaivas M. Fink J. K. (2003). The second kindred with autosomal dominant distal myopathy linked to chromosome 14q: genetic and clinical analysis. Arch. Neurol. 60, 1321–1325. 10.1001/archneur.60.9.1321 12975303

[B22] Hill A. V. (1910). The possible effects of the aggregation of the molecules of haemoglobin on its dissociation curves. J. Physiol. 40 (Suppl.), iv–vii.

[B23] Hoffmann B. Schmidt-Traub H. Perrot A. Osterziel K. J. Gessner R. (2001). First mutation in cardiac troponin C, L29Q, in a patient with hypertrophic cardiomyopathy. Hum. Mutat. 17, 524. 10.1002/humu.1143 11385718

[B24] Homayoun H. Khavandgar S. Hoover J. M. Mohsen A. W. Vockley J. Lacomis D.. (2011). Novel mutation in MYH7 gene associated with distal myopathy and cardiomyopathy. Neuromuscul. Disord. 21, 219–222. 10.1016/j.nmd.2010.12.005 21211974

[B25] Hootsmans W. J. Meerschwam I. S. (1971). Electromyography in patients with hypertrophic obstructive cardiomyopathy. Neurology 21, 810–816. 10.1212/WNL.21.8.810 5106297

[B26] Hwang P. M. Cai F. Pineda-Sanabria S. E. Corson D. C. Sykes B. D. (2014). The cardiac-specific N-terminal region of troponin I positions the regulatory domain of troponin C. Proc. Natl. Acad. Sci. U.S.A. 111, 14412–14417. 10.1073/pnas.1410775111 25246568 PMC4210035

[B27] Jaafar N. Girolami F. Zairi I. Kraiem S. Hammami M. Olivotto I. (2015). Genetic profile of hypertrophic cardiomyopathy in Tunisia: Is it different? Glob. Cardiol. Sci. Pract. 2015:16. 10.5339/gcsp.2015.16 26779504 PMC4448072

[B28] Jayasundar J. J. Xing J. Robinson J. M. Cheung H. C. Dong W. J. (2014). Molecular dynamics simulations of the cardiac troponin complex performed with FRET distances as restraints. PLoS ONE 9:e87135. 10.1371/journal.pone.0087135 24558365 PMC3928104

[B29] Karandreas N. Stathis P. Anastasakis A. Rigopoulos A. Piperos P. Theopistou A.. (2000). Electromyographic evidence of subclinical myopathy in hypertrophic cardiomyopathy. Muscle Nerve 23, 1856–1861. 10.1002/1097-4598(200012)23:12<1856::AID-MUS9>3.0.CO;2-T 11102909

[B30] Laemmli U. K. (1970). Cleavage of structural proteins during the assembly of the head of bacteriophage T4. Nature 227, 680–685. 10.1038/227680a0 5432063

[B31] Lamont P. J. Udd B. Mastaglia F. L. de Visser M. Hedera P. Voit T.. (2006). Laing early onset distal myopathy: slow myosin defect with variable abnormalities on muscle biopsy. J. Neurol. Neurosurg. Psychiatr. 77, 208–215. 10.1136/jnnp.2005.073825 16103042 PMC2077563

[B32] Lamont P. J. Wallefeld W. Hilton-Jones D. Udd B. Argov Z. Barboi A. C.. (2014). Novel mutations widen the phenotypic spectrum of slow skeletal/β-cardiac myosin (MYH7) distal myopathy. Hum. Mutat. 35, 868–879. 10.1002/humu.22553 24664454 PMC4112555

[B33] Landstrom A. P. Ackerman M. J. (2012). Beyond the cardiac myofilament: hypertrophic cardiomyopathy- associated mutations in genes that encode calcium-handling proteins. Curr. Mol. Med. 12, 507–518. 10.2174/156652412800620020 22515980 PMC3940075

[B34] Landstrom A. P. Parvatiyar M. S. Pinto J. R. Marquardt M. L. Bos J. M. Tester D. J.. (2008). Molecular and functional characterization of novel hypertrophic cardiomyopathy susceptibility mutations in TNNC1-encoded troponin C. J. Mol. Cell. Cardiol. 45, 281–288. 10.1016/j.yjmcc.2008.05.003 18572189 PMC2627482

[B35] Li M. X. Hwang P. M. (2015). Structure and function of cardiac troponin C (TNNC1): Implications for heart failure, cardiomyopathies, and troponin modulating drugs. Gene 571, 153–166. 10.1016/j.gene.2015.07.074 26232335 PMC4567495

[B36] Li Z. L. Lilienbaum A. Butler-Browne G. Paulin D. (1989). Human desmin-coding gene: complete nucleotide sequence, characterization and regulation of expression during myogenesis and development. Gene 78, 243–254. 10.1016/0378-1119(89)90227-8 2673923

[B37] Lochner A. Hewlett R. H. O'Kennedy A. van der Walt J. J. Tiedt F. A. Hoffman H.. (1981). A study of a family with inherited disease of cardiac and skeletal muscle. Part II. Skeletal muscle morphology and mitochondrial oxidative phosphorylation. S. Afr. Med. J. 59, 453–461. 6259763

[B38] Macera M. J. Szabo P. Wadgaonkar R. Siddiqui M. A. Verma R. S. (1992). Localization of the gene coding for ventricular myosin regulatory light chain (MYL2) to human chromosome 12q23-q24.3. Genomics 13, 829–831. 10.1016/0888-7543(92)90161-K 1386340

[B39] Margossian S. S. Lowey S. (1982). Preparation of myosin and its subfragments from rabbit skeletal muscle. Methods Enzymol. 85(Pt B), 55–71. 10.1016/0076-6879(82)85009-X 6214692

[B40] Marian A. J. (2010). Hypertrophic cardiomyopathy: from genetics to treatment. Eur. J. Clin. Invest. 40, 360–369. 10.1111/j.1365-2362.2010.02268.x 20503496 PMC2903630

[B41] Maron B. J. Maron M. S. (2013). Hypertrophic cardiomyopathy. Lancet 381, 242–255. 10.1016/S0140-6736(12)60397-3 22874472

[B42] Maron B. J. Maron M. S. Semsarian C. (2012). Genetics of hypertrophic cardiomyopathy after 20 years: clinical perspectives. J. Am. Coll. Cardiol. 60, 705–715. 10.1016/j.jacc.2012.02.068 22796258

[B43] Marques M. A. Pinto J. R. Moraes A. H. Iqbal A. de Magalhaes M. T. Monteiro J.. (2017). Allosteric transmission along a loosely structured backbone allows a cardiac troponin C mutant to function with only one Ca2+ ion. J. Biol. Chem. 292, 2379–2394. 10.1074/jbc.M116.765362 28049727 PMC5313108

[B44] Martins A. S. Parvatiyar M. S. Feng H. Z. Bos J. M. Gonzalez-Martinez D. Vukmirovic M.. (2015). *In vivo* analysis of troponin C knock-In (A8V) mice: evidence that TNNC1 is a hypertrophic cardiomyopathy susceptibility gene. Circ. Cardiovasc Genet. 8, 653–664. 10.1161/CIRCGENETICS.114.000957 26304555 PMC4618104

[B45] Mastaglia F. L. Lamont P. J. Laing N. G. (2005). Distal myopathies. Curr. Opin. Neurol. 18, 504–510. 10.1097/01.wco.0000175936.23945.b6 16155432

[B46] Matsuoka R. Yoshida M. C. Kanda N. Kimura M. Ozasa H. Takao A.. (1989). Human cardiac myosin heavy chain gene mapped within chromosome region 14q11.2 → q13. Am. J. Med. Genet. 32, 279–284. 10.1002/ajmg.1320320234 2494889

[B47] Meredith C. Herrmann R. Parry C. Liyanage K. Dye D. E. Durling H. J.. (2004). Mutations in the slow skeletal muscle fiber myosin heavy chain gene (MYH7) cause laing early-onset distal myopathy (MPD1). Am. J. Hum. Genet. 75, 703–708. 10.1086/424760 15322983 PMC1182058

[B48] Meyer N. L. Chase P. B. (2016). Role of cardiac troponin I carboxy terminal mobile domain and linker sequence in regulating cardiac contraction. Arch. Biochem. Biophys. 601, 80–87. 10.1016/j.abb.2016.03.010 26971468 PMC4899117

[B49] O'Connor F. G. Oriscello R. G. Levine B. D. (1999). Exercise-related syncope in the young athlete: reassurance, restriction or referral? Am. Fam. Phys. 60, 2001–2008. 10569503

[B50] Overeem S. Schelhaas H. J. Blijham P. J. Grootscholten M. I. ter Laak H. J. Timmermans J.. (2007). Symptomatic distal myopathy with cardiomyopathy due to a MYH7 mutation. Neuromuscul. Disord. 17, 490–493. 10.1016/j.nmd.2007.02.007 17383184

[B51] Parvatiyar M. S. Landstrom A. P. Figueiredo-Freitas C. Potter J. D. Ackerman M. J. Pinto J. R. (2012). A mutation in TNNC1-encoded cardiac troponin C, TNNC1-A31S, Predisposes to hypertrophic cardiomyopathy and ventricular fibrillation. J. Biol. Chem. 287, 31845–31855. 10.1074/jbc.M112.377713 22815480 PMC3442518

[B52] Pinto J. R. Gomes A. V. Jones M. A. Liang J. Nguyen S. Miller T.. (2012). The functional properties of human slow skeletal troponin T isoforms in cardiac muscle regulation. J. Biol. Chem. 287, 37362–37370. 10.1074/jbc.M112.364927 22977240 PMC3481333

[B53] Pinto J. R. Parvatiyar M. S. Jones M. A. Liang J. Ackerman M. J. Potter J. D. (2009). A functional and structural study of troponin C mutations related to hypertrophic cardiomyopathy. J. Biol. Chem. 284, 19090–19100. 10.1074/jbc.M109.007021 19439414 PMC2707221

[B54] Pinto J. R. Parvatiyar M. S. Jones M. A. Liang J. Potter J. D. (2008a). A troponin T mutation that causes infantile restrictive cardiomyopathy increases Ca2+ sensitivity of force development and impairs the inhibitory properties of troponin. J. Biol. Chem. 283, 2156–2166. 10.1074/jbc.M707066200 18032382

[B55] Pinto J. R. Reynaldo D. P. Parvatiyar M. S. Dweck D. Liang J. Jones M. A.. (2011a). Strong cross-bridges potentiate the Ca(2+) affinity changes produced by hypertrophic cardiomyopathy cardiac troponin C mutants in myofilaments: a fast kinetic approach. J. Biol. Chem. 286, 1005–1013. 10.1074/jbc.M110.168583 21056975 PMC3020707

[B56] Pinto J. R. Veltri T. Sorenson M. M. (2008b). Modulation of troponin C affinity for the thin filament by different cross-bridge states in skinned skeletal muscle fibers. Pflugers Arch. 456, 1177–1187. 10.1007/s00424-008-0480-y 18386050

[B57] Pinto J. R. Yang S. W. Hitz M. P. Parvatiyar M. S. Jones M. A. Liang J.. (2011b). Fetal cardiac troponin isoforms rescue the increased Ca2+ sensitivity produced by a novel double deletion in cardiac troponin T linked to restrictive cardiomyopathy: a clinical, genetic, and functional approach. J. Biol. Chem. 286, 20901–20912. 10.1074/jbc.M111.234336 21502316 PMC3121488

[B58] Ploski R. Rydzanicz M. Ksiazczyk T. M. Franaszczyk M. Pollak A. Kosinska J.. (2016). Evidence for troponin C (*TNNC1*) as a gene for autosomal recessive restrictive cardiomyopathy with fatal outcome in infancy. Am. J. Med. Genet. A. 170, 3241–3248. 10.1002/ajmg.a.37860 27604170

[B59] Przybojewski J. Z. Hoffman H. de Graaf A. S. van der Walt J. J. Tiedt F. A. O'Kennedy A.. (1981). A study of a family with inherited disease of cardiac and skeletal muscle. Part I. Clinical, electrocardiographic, echocardiographic, haemodynamic, electrophysiological and electron microscopic studies. S. Afr. Med. J. 59, 363–373. 7193354

[B60] Schoffstall B. LaBarbera V. A. Brunet N. M. Gavino B. J. Herring L. Heshmati S.. (2011). Interaction between troponin and myosin enhances contractile activity of myosin in cardiac muscle. DNA Cell Biol. 30, 653–659. 10.1089/dna.2010.1163 21438758 PMC3168976

[B61] Seidman C. E. Seidman J. G. (2011). Identifying sarcomere gene mutations in hypertrophic cardiomyopathy: a personal history. Circ. Res. 108, 743–750. 10.1161/circresaha.110.223834 21415408 PMC3072749

[B62] Semsarian C. Ingles J. Maron M. S. Maron B. J. (2015). New perspectives on the prevalence of hypertrophic cardiomyopathy. J. Am. Coll. Cardiol. 65, 1249–1254. 10.1016/j.jacc.2015.01.019 25814232

[B63] Smith E. R. Heffernan L. P. Sangalang V. E. Vaughan L. M. Flemington C. S. (1976). Voluntary muscle involvement in hypertrophic cardiomyopathy. A study of eleven *patients*. Ann. Intern. Med. 85, 566–572. 10.7326/0003-4819-85-5-566 988769

[B64] Solaro R. J. Pang D. C. Briggs F. N. (1971). The purification of cardiac myofibrils with Triton X-100. Biochim. Biophys. Acta 245, 259–262. 10.1016/0005-2728(71)90033-8 4332100

[B65] Song W. J. Van Keuren M. L. Drabkin H. A. Cypser J. R. Gemmill R. M. Kurnit D. M. (1996). Assignment of the human slow twitch skeletal muscle/cardiac troponin C gene (TNNC1) to human chromosome 3p21.3–>3p14.3 using somatic cell hybrids. Cytogenet. Cell Genet. 75, 36–37. 10.1159/000134453 8995486

[B66] Tajsharghi H. Oldfors A. Macleod D. P. Swash M. (2007). Homozygous mutation in MYH7 in myosin storage myopathy and cardiomyopathy. Neurology 68, 962. 10.1212/01.wnl.0000257131.13438.2c 17372140

[B67] Tajsharghi H. Thornell L. E. Lindberg C. Lindvall B. Henriksson K. G. Oldfors A. (2003). Myosin storage myopathy associated with a heterozygous missense mutation in MYH7. Ann. Neurol. 54, 494–500. 10.1002/ana.10693 14520662

[B68] Takeda S. Yamashita A. Maeda K. Maeda Y. (2003). Structure of the core domain of human cardiac troponin in the Ca(2+)-saturated form. Nature 424, 35–41. 10.1038/nature01780 12840750

[B69] Talmadge R. J. Roy R. R. (1993). Electrophoretic separation of rat skeletal muscle myosin heavy-chain isoforms. J. Appl. Physiol. 75, 2337–2340. 8307894 10.1152/jappl.1993.75.5.2337

[B70] Teare D. (1958). Asymmetrical hypertrophy of the heart in young adults. Br. Heart J. 20, 1–8. 10.1136/hrt.20.1.1 13499764 PMC492780

[B71] Towbin J. A. (2014). Inherited cardiomyopathies. Circ. J. 78, 2347–2356. 10.1253/circj.CJ-14-0893 25186923 PMC4467885

[B72] Tripet B. Van Eyk J. E. Hodges R. S. (1997). Mapping of a second actin-tropomyosin and a second troponin C binding site within the C terminus of troponin I, and their importance in the Ca2+-dependent regulation of muscle contraction. J. Mol. Biol. 271, 728–750. 10.1006/jmbi.1997.1200 9299323

[B73] Urboniene D. Dias F. A. Pena J. R. Walker L. A. Solaro R. J. Wolska B. M. (2005). Expression of slow skeletal troponin I in adult mouse heart helps to maintain the left ventricular systolic function during respiratory hypercapnia. Circ. Res. 97, 70–77. 10.1161/01.RES.0000173849.68636.1e 15961720

[B74] Vinogradova M. V. Stone D. B. Malanina G. G. Karatzaferi C. Cooke R. Mendelson R. A.. (2005). Ca^2+^-regulated structural changes in troponin. Proc. Natl. Acad. Sci. U.S.A. 102, 5038–5043. 10.1073/pnas.0408882102 15784741 PMC555973

[B75] Westfall M. V. Rust E. M. Metzger J. M. (1997). Slow skeletal troponin I gene transfer, expression, and myofilament incorporation enhances adult cardiac myocyte contractile function. Proc. Natl. Acad. Sci. U.S.A. 94, 5444–5449. 10.1073/pnas.94.10.5444 9144257 PMC24698

[B76] Weterman M. A. Barth P. G. van Spaendonck-Zwarts K. Y. Aronica E. Poll-The B. T. Brouwer O. F.. (2013). Recessive MYL2 mutations cause infantile type I muscle fibre disease and cardiomyopathy. Brain 136, 282–293. 10.1093/brain/aws293 23365102

[B77] Willott R. H. Gomes A. V. Chang A. N. Parvatiyar M. S. Pinto J. R. Potter J. D. (2010). Mutations in Troponin that cause HCM, DCM AND RCM: what can we learn about thin filament function? J. Mol. Cell. Cardiol. 48, 882–892. 10.1016/j.yjmcc.2009.10.031 19914256

[B78] Wolska B. M. Vijayan K. Arteaga G. M. Konhilas J. P. Phillips R. M. Kim R.. (2001). Expression of slow skeletal troponin I in adult transgenic mouse heart muscle reduces the force decline observed during acidic conditions. J. Physiol. 536, 863–870. 10.1111/j.1469-7793.2001.00863.x 11691878 PMC2278915

[B79] Zhang R. Zhao J. Potter J. D. (1995). Phosphorylation of both serine residues in cardiac troponin I is required to decrease the Ca2+ affinity of cardiac troponin C. J. Biol. Chem. 270, 30773–30780. 10.1074/jbc.270.51.30773 8530519

[B80] Zot H. G. Hasbun J. E. Michell C. A. Landim-Vieira M. Pinto J. R. (2016). Enhanced troponin I binding explains the functional changes produced by the hypertrophic cardiomyopathy mutation A8V of cardiac troponin C. Arch. Biochem. Biophys. 601, 97–104. 10.1016/j.abb.2016.03.011 26976709 PMC4899184

