# Supplementary material for: Hypertrophic Cardiomyopathy Cardiac Troponin C Mutations Differentially Affect Slow Skeletal and Cardiac Muscle Regulation
Source: Front Physiol. 2017 Apr 20;8:221. doi: 10.3389/fphys.2017.00221 (PMC5397416; doi:10.3389/fphys.2017.00221)
Supplement: Supplementary file 1 [file DataSheet1.pdf]

SUPPLEMENTARY MATERIAL

Hypertrophic cardiomyopathy cardiac troponin C mutations differentially affect slow skeletal and cardiac muscle regulation

Tiago Veltri[1], Maicon Landim-Vieira[1], Michelle S. Parvatiyar[2], David Gonzalez-Martinez[1], Karissa M. Dieseldorff Jones[1], Clara A. Michell[1], David Dweck[1], Andrew P. Landstrom[3], P. Bryant Chase[4], Jose R. Pinto[1]

[1]Department of Biomedical Sciences, Florida State University College of Medicine, Tallahassee, FL, USA, [2]Department of Molecular and Cellular Pharmacology, University of Miami Miller School of Medicine, Miami, FL, USA, [3]Section of Pediatric Cardiology, Department of Pediatrics, Baylor College of Medicine, Houston, TX, USA, [4]Department of Biological Science, Florida State University, Tallahassee, FL, USA

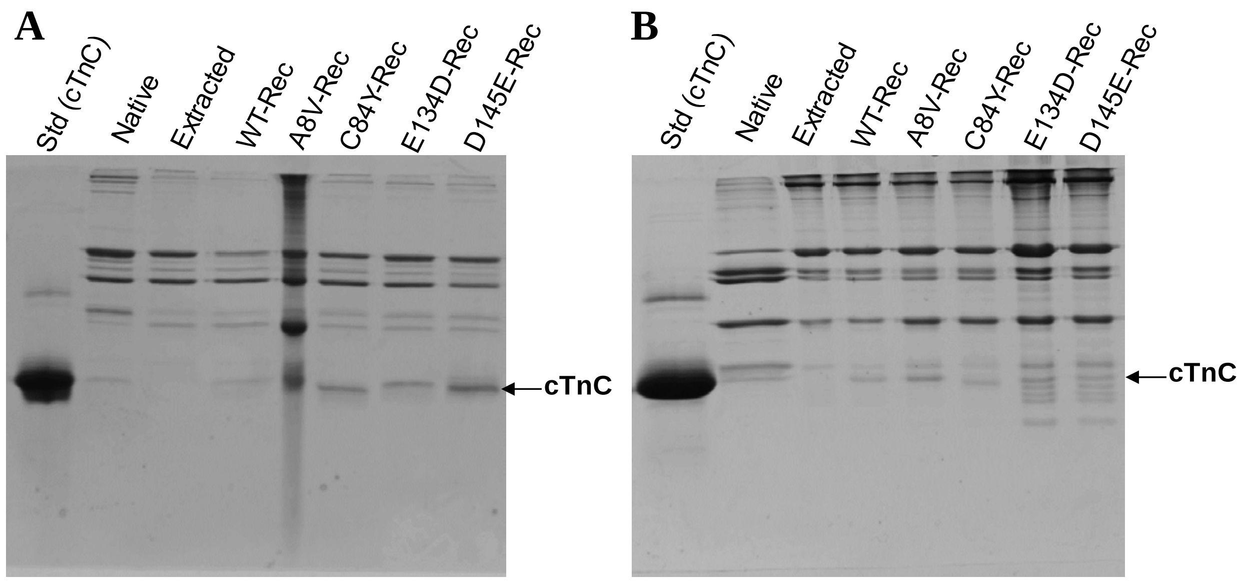

Supplementary Figure 1: 15% SDS-PAGE of native, cTnC-extracted and cTnC-reconstituted myofibrils. (A) porcine cardiac myofibrils, and (B) bovine masseter myofibrils.
